# LEAFDATA: a literature-curated database for Arabidopsis leaf development

**DOI:** 10.1186/s13007-016-0115-9

**Published:** 2016-02-15

**Authors:** Dóra Szakonyi

**Affiliations:** Instituto Gulbenkian de Ciência, 2780-156 Oeiras, Portugal

**Keywords:** Database, *Arabidopsis thaliana*, Leaf development, Manual literature curation

## Abstract

**Background:**

In the post-genomic era, biological databases provide an easy access to a wide variety of scientific data. The vast quantity of literature calls for curated databases where existing knowledge is carefully organized in order to aid novel discoveries. Leaves, the main photosynthetic organs are not only vital for plant growth but also essential for maintaining the global ecosystem by producing oxygen and food. Therefore, studying and understanding leaf formation and growth are key objectives in biology. *Arabidopsis thaliana* to this date remains the prime experimental model organism in plant science.

**Description:**

LEAFDATA was created as an easily accessible and searchable web tool to assemble a relevant collection of Arabidopsis leaf literature. LEAFDATA currently contains 13,553 categorized statements from 380 processed publications. LEAFDATA can be searched for genes of interest using Arabidopsis Genome Initiative identifiers, for selected papers by means of PubMed IDs, authors and specific keywords. The results page contains details of the original publications, text fragments from the curated literature grouped according to information types and direct links to PubMed pages of the original papers.

**Conclusions:**

The LEAFDATA database offers access to searchable entries curated from a large number of scientific publications. Due to the unprecedented details of annotations and the fact that LEAFDATA already provides records about approximately 1600 individual loci, this database is useful for the entire plant research community.

**Electronic supplementary material:**

The online version of this article (doi:10.1186/s13007-016-0115-9) contains supplementary material, which is available to authorized users.

## Background

Leaf development from primordium initiation to organ senescence is an intricate process controlled by interconnected regulatory pathways [1, 2]. Many of the key genes have been thoroughly characterized, while the role of numerous other factors with clear leaf phenotypes has not been studied in the context of leaf organogenesis. The shoot apical meristem (SAM) gives rise to the aboveground differentiated organs. The position of leaf initiation is determined by polarized auxin accumulation generated by the *YUCCA* auxin biosynthesis genes [3] and the *PIN*-*FORMED1* (*PIN1*) hormone transporter [4]. Leaf identity is established by suppression of meristem identity genes at this marked region by the MYB-family transcription factor *ASYMMETRIC LEAVES1* (*AS1*) and *AS2,* a LOB domain protein coding gene [5, 6]. A defined boundary region separates the meristem from the organ primordium and provides a border between neighboring organs. Organization of this domain depends on factors including *CUP*-*SHAPED COTYLEDON* (*CUC*) genes*, LATERAL ORGAN BOUNDARIES* (*LOB*), *LATERAL ORGAN FUSION* (*LOF1*), and *JAGGED LATERAL ORGAN* (*JLO*) genes [7]. The early leaf primordium emerges as radially symmetrical, cylindrical structure that soon differentiates along the proximodistal, mediolateral and dorsoventral axes. For the formation of a flattened leaf structure, mutually antagonistic developmental programs define the dorsal and ventral organ identity [8]. *AS1*, *AS2* and the *HD*-*ZIPIII* genes act as ventral determinants, while the *KANADI* (*KAN*) genes, the *YABBY* genes and several *AUXIN RESPONSE FACTORs* (*ARFs*) promote ventral fate. Leaf growth is a coordinated process of cell division and cell expansion. Cell divisions are driven by a great number of cell cycle regulators such as cyclins, cyclin-dependent protein kinases, and inhibitors of cyclin-dependent kinases [9]. Many of these factors are also key players in DNA endoreduplication hence crucial for controlling cell size. Cell proliferation drives early stages of leaf development, while cell expansion dominates the later phases of leaf growth. During this process, pluripotent initial cells differentiate into the abaxial and adaxial epidermis, the palisade and spongy mesophyll cell layers and the vascular system. Specific genetic and molecular pathways drive the formation of guard cells [10] and trichomes [11]. Furthermore, analysis of mutant phenotypes revealed that genes involved in chromatin remodeling, pre-mRNA splicing and processing, protein translation, post-transcriptional regulation via small RNA pathways, proteasome-dependent protein degradation, hormonal signaling, metabolite biosynthesis and numerous other processes are essential for leaf organogenesis [1, 12–16].

During the past years, several public resources have been assembled focusing on Arabidopsis leaf development. Plant morphology depends on the combination of genetic determinants and environmental factors. Regular imaging and objective measurements are crucial to monitor quantitative traits. The PHENOPSIS DB [17] is built for data storage, sharing and analysis of the precise recordings of phenotypic variables and growth conditions from automated phenotyping platforms [18, 19]. Additional measurements and offline microscopic analyses are manually added to each experiments. The database contains more than 93,000 plant images and 57,832 phenotypic details about 1057 Arabidopsis genotypes and offers data visualization and image analysis tools. The results of a systematic reverse genetic screen are summarized in PhenoLeaf [20, 21]. Approximately 24,000 SALK mutant alleles were monitored for visible leaf defects. The 706 identified leaf mutants have been cataloged and can be queried by keywords for phenotype or genes. Another collection accommodating cleared leaves with visible vascular architecture including 412 Arabidopsis pictures are available in the ClearedLeavesDB [22, 23]. The leaf senescence database (LSD) focuses on the last phase of leaf development that leads to organ death [24–26]. Manual and computational data were collected about senescence-associated genes (SAGs) from various plant species. The updated LSD 2.0 now contains 5356 genes and 322 mutants from 44 plant species, QTLs, seed information, sequence search functions and information about subcellular localization. Finally, the AGRON-OMICS consortium (Arabidopsis GROwth Network integrating OMICS technologies) was initiated to understand molecular mechanisms behind leaf growth using high-throughput experimental approaches. The effect of mild drought stress was studied in several stages of leaf development using transcript profiling and quantitative proteomics experiments [27]. These datasets along with metabolite measurements, photosynthesis and respiration rates, enzyme activities, ribosome numbers and lipid content are accessible at the project’s data integration and data sharing portal [28]. In the framework of this project a novel literature curation method was developed using the Leaf Knowtator tool and 283 key publications were processed as a community effort [29]. It was demonstrated that the collected information could be integrated with other public resources and a relational database, KnownLeaf was created. Furthermore, a graphical network was built to facilitate knowledge mining. However, access to the curated is data is hindered by the lack of a web interface. Therefore, our main aim was to establish a convenient resource with reliable query functions for easy access to this curated library.

Here, we present LEAFDATA, a high-quality and freely available literature database for Arabidopsis leaf development. By searching and manually curating 380 primary research publications, we collected 13,553 statements about genes that were experimentally linked to leaf organogenesis. We have created LEAFDATA to support fundamental research and provide a solid information resource for our users.

## Construction and content

### Data collection

LEAFDATA records were collected by employing the customized Leaf Knowtator annotation tool [29]. This interface runs in Protégé software version 3.3.1 using and the Knowtator plug-in version 1.9 beta [30]. Result sections of full-text primary research papers are processed. Entries are collected into ten major categories: phenotype, gene expression, feature, DNA–protein interaction, protein–protein interaction, genetic interaction, process, regulation of gene expression, regulation of process, and regulation of phenotype (Table 1). All categories have predefined structures and information slots attached to them that can be filled with ontology terms already uploaded into Leaf Knowtator (Table 2). The main controlled ontology collections that are included in this project are Plant Ontology (PO) [31], BRENDA Tissue Ontology (BTO) [32], Phenotype, Attribute and Trait Ontology (PATO) [33], Plant Trait Ontology (TO) [34], Molecular Interaction (MI) [35], Plant Environment Ontology (EO) [36] and Gene Ontology (GO) [37]. Genes were associated with the specific AGI identifiers derived from the TAIR10 genome annotation [38]. In addition, the Knowtator plug-in automatically saves further details such as the annotated file, annotator and annotated text. The curation system is flexible and can be easily modified to other annotation projects. Required slots are filled with terms closely following the original text. In addition to the community curations from 283 publications from the AGRON-OMICS project, 97 new papers were processed.

**Table 1:** Information types annotated in LEAFDATA

Category	Example	Number
Phenotype	The venation in each as2 leaf lamina was bilaterally asymmetrical	6559
Gene expression	YAB3 is detected in the abaxial regions of the developing leaves	4617
Feature	the AP2/EREBP domain of LEP is located close to the N-terminus of the protein	731
DNA–protein interactions	BES1 binding to the promoter of SAUR-15	151
Protein–protein interaction	AtCul1 … co-immunoprecipitated with … myc-tagged ASK1	345
Genetic interaction	se quantitatively and qualitatively enhanced the lobing of as1 … leaves	382
Process	CYCD3;1 … important for the initiation of cell division at the G1 phase in leaves	348
Regulation of gene expression	we conclude that STM negatively regulates AS1	171
Regulation of process	WRKY53, is an important positive regulator of senescence	206
Regulation of phenotype	NEK6 … promotes biomass levels	42

Ten major classes of information are curated in our database. Examples of these categories and number of statements are shown here

**Table 2:** Phenotype annotation exported from Leaf Knowtator

Database columns	Entries
File	21401745.txt.knowtator.xml
Class	Phenotype
Annotator	Dora Szakonyi, LEAFDATA
Spanned text	The rid2-1 mutant was temperature sensitive for seedling growth as well as for callus formation. In rid2-1 seedlings grown at 22 °C, the true leaves were pointed
Annotated text	Genotype ID = rid2-1|Property Slot = NULL|Value ID = pointed|Plant part ID = leaves
Growth condition	
Developmental stage	
Plant part	leaf_PO:0025034
Localisation	
Property	shape_PATO:0000052
Process	
Value	pointed_PATO:0002258
Regulation	
Gene expression	
Gene studied	
Interaction type	
Protein studied	
Interactor protein	
Protein	
Gene target	
Genetic interactor	
DNA target	
Genotype ID	mutated gene_MI:0804
Genotype details	Gene ID = AT5G57280 | Genotype_Zygosity = homozygous diploid _APO:0000229 | Mutant LOF_GOF ID = loss of function_APO:0000011
Factuality	

In LEAFDATA, a complex range of information is attached to each displayed statements deposited as spanned text. Different classes have distinct predefined slots however records are converted into a single table. Database columns corresponds to all available Leaf Knowtator slots. File, Annotator, Spanned text and Annotated text information is automatically added to curated statements

### Database construction

Annotations were exported as XML files from Leaf Knowtator. These files are small and easy to share. The XML files were transformed into a single table with a custom made Perl script [24] and loaded in bulk using Structured Query Language (SQL) queries. The LEAFDATA Database resides on the MS SQL Server 2008 platform. The website design is fully responsive in line with current industry standards and is based on the Bootstrap Framework. Bootstrap utilizes HTML, CSS, and JS frameworks for developing responsive projects on the web. For database integration the server side engine Adobe Coldfusion 9 running over MS IIS was chosen for its relatively inexpensive hosting costs, its rapid development credentials and powerful data collation functions. The employment of these cutting-edge technologies offers a modern, literature-curated website that can be used on any device and provide fast access to our data in any research environment.

## Utility

### LEAFDATA home

**Fig. 1:**
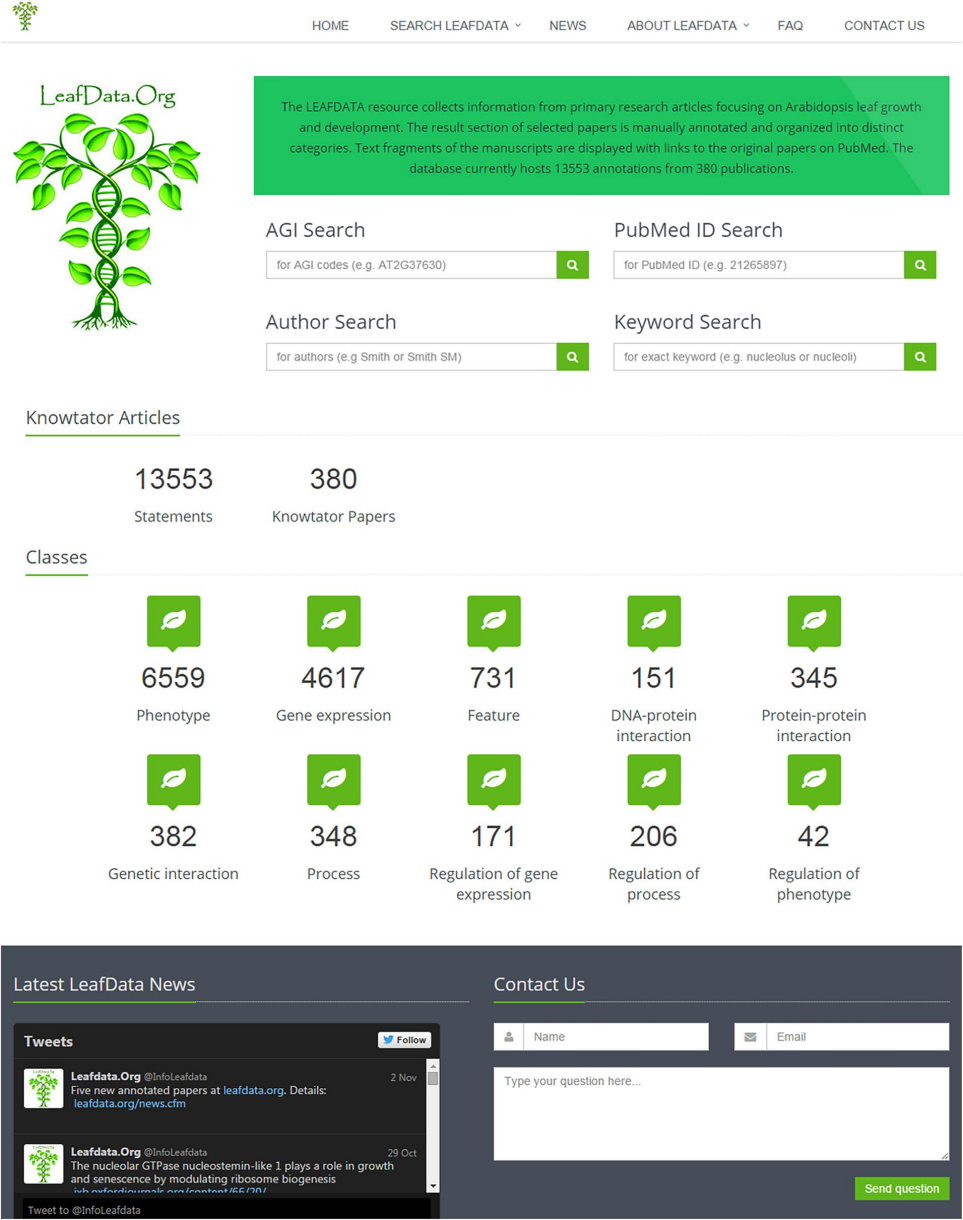
LEAFDATA home. Four key search tools, a summary of the database content, a news section connected to an active Twitter account and a contact form can be reached from the LEAFDATA main page

The main site (Fig. 1) provides direct access to the search functions. There is a visual representation of the database content including number of curated publications and individual statements, and details of the different categories. Upon selecting any categories, all annotations can be retrieved. On the bottom of the page, a news section can be found directly connected to an active Twitter account with announcements of relevant publications and database updates. Moreover, a direct contact form is available for any enquiries.

### LEAFDATA search tools

**Fig. 2:**
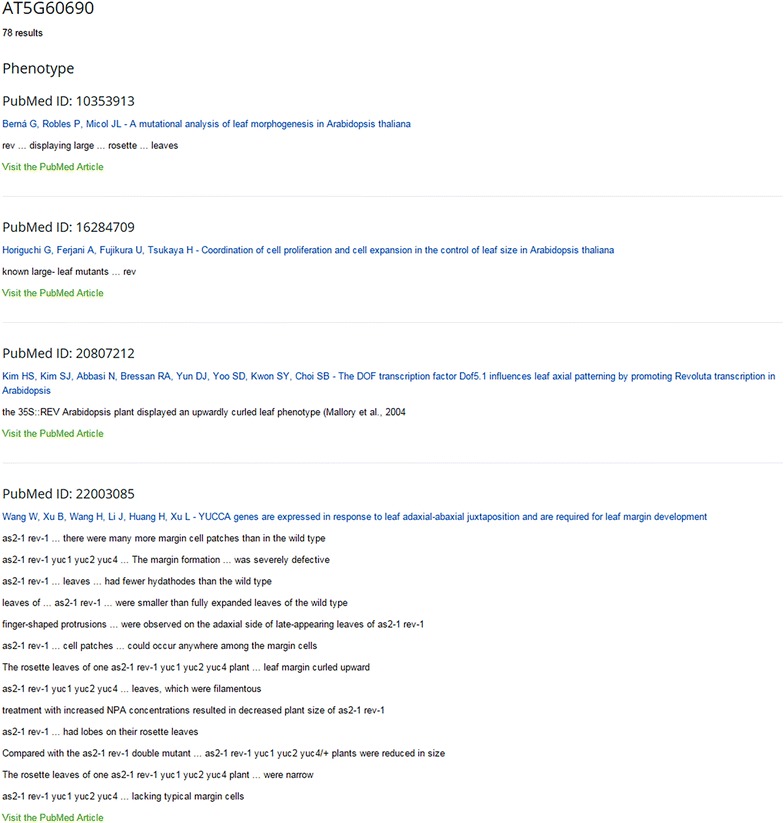
LEAFDATA result page. AGI search for AT5G60690 was performed. Records are organized according to information types and publications with direct links to the PubMed collection

LEAFDATA provides four convenient search functions. Genes of interest can be queried by using unique AGI identifiers based on the last TAIR10 genome release. All annotations can be retrieved from a selected publication using the PubMed ID. In addition to an author query, we also offer a keyword search. Results are arranged according to distinct categories and individual publications. For illustration, records from an AGI search for the *HD-ZIPIII* transcription factor *REVOLUTA* (*REV*) is shown (Fig. 2; Table 3). This query resulted in 78 statements from 17 different papers. The keyword tool is particularly helpful to attain required information. It allows combining multiple keywords and limits the search results to only those documents that contain all the terms. This function can be used effectively to find plant lines that share a certain phenotype, genes with the same biological function or similar expression domains. Recent publications revealed that genetic combinations of plant lines with increased leaf size can further enhance growth [39]. In order to find all the large-leaf Arabidopsis lines curated in LEAFDATA, we performed a search for the terms size_PATO:0000586 and increased size_PATO:0000117 and retrieved a preliminary list of 173 statements (Additional file 1). Ontology terms were used to minimize the recovery of false positive records and ‘plant part’ was not specified to maximize the number of genuine hits. Terms with similar meanings can be used for this query. For example, large leaves, big leaves, increased leaf size gave 162, 12, and 373 results, respectively (Additional file 2: Table S1). Ten statements were randomly selected for additional data mining (Table 4). First AGI codes were collected from the LEAFDATA gene list available under the SEARCH LEAFDATA tab (see also Additional File 3: Table S2) then AGI searches were performed for the individual genes. Further analysis was focused on gene expression data in wild-type background and reported biological functions. For eight genes, both gene expression and functional records were recovered. In one case, only gene expression data was found while for a sole example none of the required additional information was available in LEAFDATA. Importantly, half of these records were gathered from multiple (2–4) papers.

**Table 3:** Results using AGI search for AT5G60690

*Phenotype*
PubMed ID: 10353913 Berná G, Robles P, Micol JL—A mutational analysis of leaf morphogenesis in Arabidopsis thaliana rev … displaying large … rosette … leaves
PubMed ID: 16284709 Horiguchi G, Ferjani A, Fujikura U, Tsukaya H—Coordination of cell proliferation and cell expansion in the control of leaf size in Arabidopsis thaliana known large-leaf mutants … rev
PubMed ID: 20807212 Kim HS, Kim SJ, Abbasi N, Bressan RA, Yun DJ, Yoo SD, Kwon SY, Choi SB—The DOF transcription factor Dof5.1 influences leaf axial patterning by promoting Revoluta transcription in Arabidopsis the 35S::REV Arabidopsis plant displayed an upwardly curled leaf phenotype (Mallory et al. 2004)
PubMed ID: 22003085 Wang W, Xu B, Wang H, Li J, Huang H, Xu L—YUCCA genes are expressed in response to leaf adaxial-abaxial juxtaposition and are required for leaf margin development as2-1 rev-1 … there were many more margin cell patches than in the wild type as2-1 rev-1 yuc1 yuc2 yuc4 … The margin formation … was severely defective as2-1 rev-1 … leaves … had fewer hydathodes than the wild type leaves of … as2-1 rev-1 … were smaller than fully expanded leaves of the wild type finger-shaped protrusions … were observed on the adaxial side of late-appearing leaves of as2-1 rev-1 as2-1 rev-1 … cell patches … could occur anywhere among the margin cells The rosette leaves of one as2-1 rev-1 yuc1 yuc2 yuc4 plant … leaf margin curled upward as2-1 rev-1 yuc1 yuc2 yuc4 … leaves, which were filamentous treatment with increased NPA concentrations resulted in decreased plant size of as2-1 rev-1 as2-1 rev-1 … had lobes on their rosette leaves Compared with the as2-1 rev-1 double mutant … as2-1 rev-1 yuc1 yuc2 yuc4/+ plants were reduced in size The rosette leaves of one as2-1 rev-1 yuc1 yuc2 yuc4 plant … were narrow as2-1 rev-1 yuc1 yuc2 yuc4 … lacking typical margin cells
PubMed ID: 7555701 Talbert PB, Adler HT, Parks DW, Comai L—The REVOLUTA gene is necessary for apical meristem development and for limiting cell divisions in the leaves and stems of Arabidopsis thaliana leaves of rev-1 mutants grew abnormally large The rosette leaves of rev-1 plants were not readily distinguishable from wild-type No-0 leaves prior to bolting. As bolting began, however, the youngest rosette leaves became abnormally large … as they matured The rev-1 mutation caused overgrowth of both rosette and cauline leaves The rosette leaves of rev-1 plants were not readily distinguishable from wild-type No-0 leaves prior to bolting. As bolting began, however, the youngest rosette leaves became … distorted or uneven in shape as they matured mutants with a syndrome con- sisting of revolute (downwardly curled) leaves … rev-1, rev-2 and rev-4 Both the leaves and the primary shoots of rev-1 mutants were often darker green than those of wild type The difference in leaf size between wild-type and rev-1 plants was not obvious in the earlier rosette leaves, but we measured significant size differences in the cotyledons and first and third leaves from cohort 3 (Table 2). Later leaves differed more dramatically: the mean length of the longest rosette leaf (ordinarily the youngest leaf) of rev-1 plants was about 39 % longer than wild-type controls, and rev-1 cauline leaves became up to twice as long as their wild-type counterparts
*Gene expression*
PubMed ID: 11525739 Eshed Y, Baum SF, Perea JV, Bowman JL—Establishment of polarity in lateral organs of plants mRNA of REV … Later expression is confined to the provascular and vascular tissues of leaves mRNA of REV … restricted to the adaxial domain as developing primordia REV … initiates normally in kan1 kan2 leaf primordia mRNA of REV … is localized to the SAM REV … in kan1 kan2 leaf primordia … confinement to the adaxial domain is delayed mRNA of REV … is localized to the SAM, throughout leaf primordia anlagen
PubMed ID: 12615938 Nelissen H, Clarke JH, De Block M, De Block S, Vanderhaeghen R, Zielinski RE, Dyer T, Lust S, Inzé D, Van Lijsebettens M—DRL1, a homolog of the yeast TOT4/KTI12 protein, has a function in meristem activity and organ growth in plants pREV(OLUTA)-GUS … were introgressed into drl1-2 … marker lines displayed promoter activity in the dorsal part of the leaf primordium, including the vascular bundles
PubMed ID: 16682355 Garcia D, Collier SA, Byrne ME, Martienssen RA—Specification of leaf polarity in Arabidopsis via the trans-acting siRNA pathway REVOLUTA … Expression is re- stricted to the adaxial domain
PubMed ID: 16699177 Xu L, Yang L, Pi L, Liu Q, Ling Q, Wang H, Poethig RS, Huang H—Genetic interaction between the AS1-AS2 and RDR6-SGS3-AGO7 pathways for leaf morphogenesis leaves of the sgs3-11 as2-101 … contained … reduced levels of REV transcripts REV transcripts were detected in the earlier stage leaf primordia in … zip as1-101 leaves of the … zip as2-101 … contained … reduced levels of REV transcripts REV … repressed … in the rdr6-3 as2-101 leaves REV transcripts were detected in the earlier stage leaf primordia in … sgs3-11 as2-101 rdr6-3 as2-101 … leaves … contained … reduced levels of REV transcripts REV transcripts were detected in the earlier stage leaf primordia in … zip as2-101 REV transcripts were detected in the earlier stage leaf primordia in sgs3-11 as1-101
PubMed ID: 17559509 Iwakawa H, Iwasaki M, Kojima S, Ueno Y, Soma T, Tanaka H, Semiarti E, Machida Y, Machida C—Expression of the ASYMMETRIC LEAVES2 gene in the adaxial domain of Arabidopsis leaves represses cell proliferation in this domain and is critical for the development of properly expanded leaves in as2-1 … No significant differences in levels of transcripts were detected for … REV in as1-1 … No significant differences in levels of transcripts were detected for … REV
PubMed ID: 19717616 Vandenbussche M, Horstman A, Zethof J, Koes R, Rijpkema AS, Gerats T—Differential recruitment of WOX transcription factors for lateral development and organ fusion in Petunia and Arabidopsis In Arabidopsis, organ polarity regulation has been characterized extensively at the molecular level. We therefore have monitored the expression levels of a selection of Arabidopsis genes known to be involved in abaxial/adaxial patterning. The selection of genes comprises … REVOLUTA … None of the monitored Arabidopsis genes exhibited significant changes in transcript levels between wox1 prs mutant samples and the wild type
PubMed ID: 20628155 Sarojam R, Sappl PG, Goldshmidt A, Efroni I, Floyd SK, Eshed Y, Bowman JL—Differentiating Arabidopsis shoots from leaves by combined YABBY activities YABBY triple mutants display reduced expression of … REV
PubMed ID: 20807212 Kim HS, Kim SJ, Abbasi N, Bressan RA, Yun DJ, Yoo SD, Kwon SY, Choi SB—The DOF transcription factor Dof5.1 influences leaf axial patterning by promoting Revoluta transcription in Arabidopsis REV and ATHB-15 trans-cripts were strongly enhanced in Dof5.1-D (Figure 6a); however, ATHB-15 was not increased in DEX::Dof5.1 plants upon DEX treatment whereas REV transcript was enhanced (Figure 6b). Increased expression of ATHB-15 in Dof5.1-D is probably due to a secondary effect REV expression was decreased in 35S::Dof5.1ΔAct plants although the levels were different depending on transgenic lines
PubMed ID: 21223391 Szakonyi D, Byrne ME—Ribosomal protein L27a is required for growth and patterning in Arabidopsis thaliana REV:REV-VENUS … expressed in the apical and central regions of wild-type embryos
PubMed ID: 21251100 Horiguchi G, Mollá-Morales A, Pérez-Pérez JM, Kojima K, Robles P, Ponce MR, Micol JL, Tsukaya H—Differential contributions of ribosomal protein genes to Arabidopsis thaliana leaf development agreement with the synergistic polarity defects observed in rpl4d-3 as2-1 … expression … REV were expressed at similar levels in the … parents
PubMed ID: 22003085 Wang W, Xu B, Wang H, Li J, Huang H, Xu L—YUCCA genes are expressed in response to leaf adaxial-abaxial juxtaposition and are required for leaf margin development pDR5::GUS … staining in the as2-1 rev-1 … leaves was also concentrated in the top portion of the protrusions YUC4 was expressed in the leaf protrusions of the mock-treated as2-1 rev-1 as2-1 rev-1 … when … treated with 1 μM NPA … we did not observe the small GUS-staining spots on leaf surfaces as2-1 rev-1 … expressions of … YUC4 … associated with the protrusions on leaves as2-1 rev-1 … expressions of YUC1 … associated with the protrusions on leaves as2-1 rev-1 … expressions of … YUC2 … associated with the protrusions on leaves
PubMed ID: 22026817 Xu D, Huang W, Li Y, Wang H, Huang H, Cui X—Elongator complex is critical for cell cycle progression and leaf patterning in Arabidopsis We next examined expression of leaf polarity marker genes REVOLUTA (REV) and FILAMENTOUS FLOWER (FIL) in the elo2 as2 background. rev-9 is a T-DNA enhancer trap line in which β-glucuronidase (GUS) staining represents the expression of the leaf adaxial marker REV (Emery et al. 2003; Hawker and Bowman 2004). Compared with that in the rev-9/+ plant (Fig. 1q), GUS staining was not detected from needle-like leaves of the elo2 as2 rev-9/+ plant
PubMed ID: 23268445 Ben Chaabane S, Liu R, Chinnusamy V, Kwon Y, Park JH, Kim SY, Zhu JK, Yang SW, Lee BH.—STA1, an Arabidopsis pre-mRNA processing factor 6 homolog, is a new player involved in miRNA biogenesis Compared with WT, the accumulation of … REV transcripts was higher in sta1-1, which is linked to decreased miR164/166 levels and explains the serrated leaf phenotype of sta1-1
PubMed ID: 24464295 Huang T, Harrar Y, Lin C, Reinhart B, Newell NR, Talavera-Rauh F, Hokin SA, Barton MK, Kerstetter RA—Arabidopsis KANADI1 acts as a transcriptional repressor by interacting with a specific cis-element and regulates auxin biosynthesis, transport, and signaling in opposition to HD-ZIPIII factors we found evidence for regulation by … GR-REV of … At5g47800 A third technique, qRT-PCR, on independent samples confirmed statistically significant upregulation of NPY1 by GR-REV in the presence and absence of CHX, indicating that NPY1 is likely a direct target of REV activation qRT-PCR on an independent set of samples showed upregulation of WAG1 by GR-REV both in the presence and absence of CHX we found evidence for regulation by … GR-REV of … ARF3 we found evidence for regulation by … GR-REV of … LAX3 we found evidence for regulation by … GR-REV of … YUCCA5 we found evidence for regulation by … GR-REV of … LAX1 we found evidence for regulation by … GR-REV of … At1g50280 we found evidence for regulation by … GR-REV of … LAX2 we found evidence for regulation by … GR-REV of … TAA1 we found evidence for regulation by … GR-REV of … ENP1/NPY1 ARF3/ETTIN showed reproducible upregulation by GR-REV we found evidence for regulation by … GR-REV of … At1g52770
*DNA–protein interaction*
PubMed ID: 20807212 Kim HS, Kim SJ, Abbasi N, Bressan RA, Yun DJ, Yoo SD, Kwon SY, Choi SB—The DOF transcription factor Dof5.1 influences leaf axial patterning by promoting Revoluta transcription in Arabidopsis The results from both in vitro and in vivo binding assays demonstrate that Dof5.1 directly binds to the REV promoter The EMSA result showed that, GST alone did not bind to the 89-bp long substrate (Figure 6d left, lane 2) but the GST–Dof5.1DB fusion protein migrated with promoter DNA
*Genetic interaction*
PubMed ID: 20807212 Kim HS, Kim SJ, Abbasi N, Bressan RA, Yun DJ, Yoo SD, Kwon SY, Choi SB—The DOF transcription factor Dof5.1 influences leaf axial patterning by promoting Revoluta transcription in Arabidopsis The resulting Dof5.1-D/rev plants lacked the upward-curling phenotype of Dof5.1-D, thereby displaying almost WT morphology
PubMed ID: 22003085 Wang W, Xu B, Wang H, Li J, Huang H, Xu L—YUCCA genes are expressed in response to leaf adaxial-abaxial juxtaposition and are required for leaf margin development leaves … as2-1 rev-1 … were smaller than … those of the corresponding single mutants
*Process*
PubMed ID: 16682355 Garcia D, Collier SA, Byrne ME, Martienssen RA—Specification of leaf polarity in Arabidopsis via the trans-acting siRNA pathway REVOLUTA … specifying adaxial identity
*Regulation of gene expression*
PubMed ID: 16682355 Garcia D, Collier SA, Byrne ME, Martienssen RA—Specification of leaf polarity in Arabidopsis via the trans-acting siRNA pathway The microRNA miR165, which regulates class III HD- ZIP gene expression through transcript cleavage REVOLUTA … Expression is re- stricted … by KANADI (KAN) genes
PubMed ID: 20807212 Kim HS, Kim SJ, Abbasi N, Bressan RA, Yun DJ, Yoo SD, Kwon SY, Choi SB—The DOF transcription factor Dof5.1 influences leaf axial patterning by promoting Revoluta transcription in Arabidopsis These results strongly indicate that Dof5.1 activates REV transcription
PubMed ID: 24464295 Huang T, Harrar Y, Lin C, Reinhart B, Newell NR, Talavera-Rauh F, Hokin SA, Barton MK, Kerstetter RA—Arabidopsis KANADI1 acts as a transcriptional repressor by interacting with a specific cis-element and regulates auxin biosynthesis, transport, and signaling in opposition to HD-ZIPIII factors REV increases transcription, most likely by direct activation, of NPY1
*Regulation of phenotype*
PubMed ID: 16682355 Garcia D, Collier SA, Byrne ME, Martienssen RA—Specification of leaf polarity in Arabidopsis via the trans-acting siRNA pathway REVOLUTA, influence leaf shape

Seventy-eight records are available in LEAFDATA for AT5G60690, REV gene. These statements are organized according to information type and original publication

**Table 4:** Mining LEAFDATA for increased leaf size phenotype

AGI	Phenotype	Gene expression	Process
AT4G36380	The rot3-2 allele causes enlarged leaf blades (10430960)	Leaves, epidermis, palisade tissue, and the spongy layer (10430960)	Cell elongation (11889033, 9694802) Elongation of leaves (10430960) Longitudinal cell expansion (17038516)
AT1G56010	35S::NAC1 overexpressing lines … were bigger (11114891)	Leaf primordia, nucleus, low levels in leaves (11114891)	transcriptional activator (11114891)
AT3G59900	35S-ARGOS … lines showed an enlarged … leaf size (12566580)	Young rosette leaves, juvenile leaf … petioles, juvenile leaf blades, cytosol, nucleus, leaf primordia (12953103) Juvenile leaves (16824178) ER-localized (21457262)	Controls later organ growth by affecting the duration of cell proliferation (16824178)
AT5G62000	Homozygous plants of the arf2-6 … have … large … rosette leaves (15960614)	ARF2 is expressed in all major plant organs including roots, rosette and cauline leaves, flowers and siliques (15960614, 16339187)	Leaf development during leaf expansion (16176952) Repressor (18599455) Negative regulator of the BR pathway (18599455)
AT3G13960	AtGRF5 overexpressers … developed leaves that were 20–30 % larger than those of the wild type (15960617)	Low in mature stems and leaves, shoot tips containing the shoot apical meristem (SAM) (12974814) Primordium, restricted to the lower half of the leaf primordium, undetectable in mature leaves (15960617)	Promoting … cell proliferation, promoting leaf growth (15960617)
AT1G17110	UBP15 over-expression lines revealed larger overall stature of the plants as well as larger rosette leaves (18485060)	Higher in rosette leaves, increased from the early to late leaf stages, with higher expression in the leaf margin in the late stage, present in both the cytosol and nucleus (18485060)	De-ubiquitinating enzyme (18485060)
AT4G18390	TCP2 … Loss-of-function … had … slightly enlarged leaves (18816164)		
AT4G22270	AtMRB1 overexpressor plants … exhibited enlarged organ sizes (19200151)	Shoot tips and shoot apical meristems (SAM), young leaves (19200151)	
AT4G29040	rpt2a-2 mutant … displayed a phenotype of enlarged rosette leaves (19500299)	SAM, all the organs that we tested (flower bud, stem, leaf and root) (15073153) All organs tested, trichomes, expanded cotyledons, vascular cells, shoot meristem (19500299)	Proteasome activity (15073153)
AT3G44200	At the flowering stage, the two NEK6-overexpressing lines exhibited … larger rosette than Col (21801253)	Leaves, young leaves, mature rosette leaves, vascular tissues, petioles (21801253)	Stress response, rosette growth, suppresses expression of several ethylene biosynthesis (21801253)

Ten representative phenotype records were chosen from the keyword query for the terms size_PATO:0000586 and increased size_PATO:0000117. Subsequently, gene expression, process and regulation of biological process statements were collected from specific AGI searches. PubMed IDs of the parent publications are shown in brackets

All the query tools can be accessed from the main site as well as from dedicated search pages where queries can be restricted to different categories. Finally, to show the full content of LEAFDATA, there is a current list of all annotated papers under the SEARCH LEAFDATA tab (Additional File 4: Table S3).

## Discussion

Leaves are essential organs for plant life and the location of multiple biological processes. Organogenesis from emergence of leaf primordium through pattern formation, maturation, maintenance until senescence is regulated by diverse regulatory pathways. Genetic and molecular roles of numerous genes were described in great detail. These genes are classified as key players in leaf morphogenesis. However, numerous additional genes causing altered leaf morphology have been isolated. In many cases, characterization of the observed leaf phenotypes are not main scope of these studies. Furthermore, these information are scattered throughout the existing scientific literature. Our aim was to create a convenient public collection of relevant leaf literature that provides simple query functions and easy access to a large library at the same time. Here, we demonstrate that our published annotation method and the Leaf Knowtator interface [29] can be used effectively for establishing high-quality literature resources. Employing this system guaranteed several unique database features. With a quick workflow, we are able to retain a large amount of information. In LEAFDATA, not only are the curated text fragments from the original publications kept and displayed but ontology terms from established structured vocabularies are simultaneously attached to these statements. Using these standardized terms helps building complex queries and can facilitate data sharing and integration [40]. We adhere to further community standards by employing the entity–attribute–value (EAV) model for phenotype annotations [41]. On average, more than 35 annotations per publication are generated adding up to a total of 13,553 independent statements about nearly 1300 genes. A major advantage of our database is that our curations are not restricted to single genotypes or information types. For instance, phenotype annotations can cover descriptions of single and multiple mutants (Table 3) as well as constitutive or inducible overexpressors, transgenic plants expressing chimeric constructs or modified versions of the gene of interest. Also, gene expression records provide an exceptional range of information including quantification of expression levels and spatial distribution in wild type or various mutant backgrounds (Table 3). Most of our annotations belong to the phenotype and gene expression class however numerous protein–protein interaction, genetic interaction and DNA–protein interaction records can be accessed (Table 1). The original publication details (author, title, PubMed ID) are clearly displayed with each statements and a direct link is provided to the dedicated PubMed page. The search functions were designed to give a quick access to records from a chosen gene, paper or author. The keyword query allows more detailed data mining e.g. for a specific genotype using multiple terms. In summary, the combination of the LEAFDATA tools can be used effectively to collect wide-range of information (Table 4).

LEAFDATA is a useful platform not only for researchers interested in leaf development but for scientists working with other traits, plant species or model organisms. There are possible applications for our dataset in large-scale projects, mutagenesis screens and developing text-mining tools. University students, interested professionals and the general public can benefit from free and easy access to the LEAFDATA library offering processed scientific records.

We envision future improvements for LEAFDATA. The current database contains approximately 15–20 % of the published Arabidopsis leaf literature, is constantly being updated. However, it will take significant effort to annotate every existing leaf development paper and at the same time keep up with the steady flow of new research. We plan to develop advanced search functions for instance queries for specific phenotypic characteristics, combinations of features or exclusion certain traits. Similarly, gene expression statements can be further explored by genotypes, changes in certain target genes or expression in special subcellular compartments, cell types and organs. Lastly, we are interested in data visualization and integration with other datasets.

## Conclusions

The sheer amount of scientific literature is calling for carefully curated database summarizing experimental results. We employed the Leaf Knowtator curation system and constructed a unique, comprehensive database focusing on Arabidopsis leaf development. In addition to previously described regulators, genes with clear leaf phenotypes are included. The LEAFDATA collection gives access to 380 publications organized according to papers and information types. Four query functions provide easy access to high-quality annotations and direct links to the original papers. LEAFDATA serves as a valuable resource and reference point for the research community. Finally, our annotation approach, data organization and database structure can serve as a prototype for other literature curation projects.

## Availability and requirements

LEAFDATA is an open access database at www.leafdata.org. The collection is updated on a regular basis. Questions, comments and requests regarding this database should be sent to Dóra Szakonyi at info@leafdata.org.

Details of LEAFDATA content and screenshots were recorded on 08/11/2015.

## Additional files

10.1186/s13007-016-0115-9 Results using keyword search for size_PATO:0000586 increased size_PATO:0000117. A preliminary list of 173 results were recovered. Confirmed records are highlighted in yellow.
10.1186/s13007-016-0115-9 Number of results retrieved by keywords search using different terms for large-leaf phenotype.
10.1186/s13007-016-0115-9 List of genes curated in LEAFDATA.
10.1186/s13007-016-0115-9 List of papers curated in LEAFDATA.
