# Supplementary material for: LEAFDATA: a literature-curated database for Arabidopsis leaf development
Source: Plant Methods. 2016 Feb 15;12:15. doi: 10.1186/s13007-016-0115-9 (PMC4754890; doi:10.1186/s13007-016-0115-9)
Supplement: Supplementary file 1 — 10.1186/s13007-016-0115-9 Results using keyword search for size_PATO:0000586 increased size_PATO:0000117. A preliminary list of 173 results were recovered. Confirmed records are highlighted in yellow. [file 13007_2016_115_MOESM1_ESM.docx]

**Additional file 1 Results using keyword search for size_PATO:0000586 increased size_PATO:0000117.** A preliminary list of 173 results were recovered. Confirmed records are highlighted in yellow.

**size_PATO:0000586 increased size_PATO:0000117**

173 results

**Phenotype**

**PubMed ID: 10353913**

Berná G, Robles P, Micol JL - A mutational analysis of leaf morphogenesis in Arabidopsis thaliana

rev ... displaying large ... rosette ... leaves

[Visit the PubMed Article](http://www.ncbi.nlm.nih.gov/pubmed/10353913)

**PubMed ID: 10430960**

Kim GT, Tsukaya H, Saito Y, Uchimiya H - Changes in the shapes of leaves and flowers upon overexpression of cytochrome P450 in Arabidopsis

When we induced the expression of a rot3-2 allele, ROT3G80E, into a null-type rot3-1 parent, plants had slightly enlarged leaf blades

The rot3-2 allele causes enlarged leaf blades

[Visit the PubMed Article](http://www.ncbi.nlm.nih.gov/pubmed/10430960)

**PubMed ID: 10639184**

Mizukami Y, Fischer RL - Plant organ size control: AINTEGUMENTA regulates growth and cell numbers during organogenesis

plants highly expressed the ANT transgene ... leaves ... were dramatically enlarged

[Visit the PubMed Article](http://www.ncbi.nlm.nih.gov/pubmed/10639184)

**PubMed ID: 10931949**

Cho HT, Cosgrove DJ - Altered expression of expansin modulates leaf growth and pedicel abscission in Arabidopsis thaliana

AtEXP10 ... sense ... lines, rosette size became greater than that in controls after day 30, which is when the largest rosette leaves started to reach full size

AtEXP10 ... sense plants were slightly larger and matured earlier than did control plants

[Visit the PubMed Article](http://www.ncbi.nlm.nih.gov/pubmed/10931949)

**PubMed ID: 11044410**

van der Graaff E, Dulk-Ras AD, Hooykaas PJ, Keller B - Activation tagging of the LEAFY PETIOLE gene affects leaf petiole development in Arabidopsis thaliana

To study the effect of constitutive overexpression of LEP on plant development, the coding region of LEP was amplified by ... PCR and cloned in a 35S overexpression cassette ... The cDNAoe-B plants formed several rosette leaves but they remained small and the plants looked dwarfed

let leaves ... the leaf blade was larger

[Visit the PubMed Article](http://www.ncbi.nlm.nih.gov/pubmed/11044410)

**PubMed ID: 11114891**

Xie Q, Frugis G, Colgan D, Chua NH - Arabidopsis NAC1 transduces auxin signal downstream of TIR1 to promote lateral root development

35S::NAC1 overexpressing lines ... were bigger

35S::NAC1 overexpressing lines ... were ... with larger leaves

[Visit the PubMed Article](http://www.ncbi.nlm.nih.gov/pubmed/11114891)

**PubMed ID: 12426376**

Autran D, Jonak C, Belcram K, Beemster GT, Kronenberger J, Grandjean O, Inzé D, Traas J - Cell numbers and leaf development in Arabidopsis: a functional analysis of the STRUWWELPETER gene

swp leaves ... majority of the pavement cells ... contained larger nuclei

swp leaves ... some of the mesophyll cells contained larger nuclei

[Visit the PubMed Article](http://www.ncbi.nlm.nih.gov/pubmed/12426376)

**PubMed ID: 12509523**

Dewitte W, Riou-Khamlichi C, Scofield S, Healy JM, Jacqmard A, Kilby NJ, Murray JA - Altered cell cycle distribution, hyperplasia, and inhibited differentiation in Arabidopsis caused by the D-type cyclin CYCD3

Because cotyledons of CYCD3;1 OE plants were larger than those in wild-type plants

CaMV35S-CYCD3;1 ... In young seedlings, cotyledons were enlarged

[Visit the PubMed Article](http://www.ncbi.nlm.nih.gov/pubmed/12509523)

**PubMed ID: 12566580**

Ullah H, Chen JG, Temple B, Boyes DC, Alonso JM, Davis KR, Ecker JR, Jones AM - The beta-subunit of the Arabidopsis G protein negatively regulates auxin-induced cell division and affects multiple developmental processes

agb1 seedlings are larger ... than wild-type seedlings

[Visit the PubMed Article](http://www.ncbi.nlm.nih.gov/pubmed/12566580)

**PubMed ID: 12615942**

Kirch T, Simon R, Grünewald M, Werr W - The DORNROSCHEN/ENHANCER OF SHOOT REGENERATION1 gene of Arabidopsis acts in the control of meristem ccll fate and lateral organ development

DRN/ESR1 ... coding region under the control of the CaMV35S promoter in transgenic plants ... second class comprising 22 plantlets ... Sections through seedlings showed an enlarged ... SAM

[Visit the PubMed Article](http://www.ncbi.nlm.nih.gov/pubmed/12615942)

**PubMed ID: 12620976**

Bollman KM, Aukerman MJ, Park MY, Hunter C, Berardini TZ, Poethig RS - HASTY, the Arabidopsis ortholog of exportin 5/MSN5, regulates phase change and morphogenesis

3-day-old hst-1 seedlings (Fig. 2F) have a larger ... SAM

[Visit the PubMed Article](http://www.ncbi.nlm.nih.gov/pubmed/12620976)

**PubMed ID: 12953103**

Hu Y, Xie Q, Chua NH - The Arabidopsis auxin-inducible gene ARGOS controls lateral organ size

35S-ARGOS ... lines showed an enlarged ... leaf size

overexpression of ARGOS ... axr1-3 ... producing similarly sized or even larger leaves compared with wild-type leaves

[Visit the PubMed Article](http://www.ncbi.nlm.nih.gov/pubmed/12953103)

**PubMed ID: 12974814**

Kim JH, Choi D, Kende H - The AtGRF family of putative transcription factors is involved in leaf and cotyledon growth in Arabidopsis

AtGRF1 4-7 ... overexpressors ... developed larger ... cotyledons

AtGRF2 7-2 ... overexpressors ... developed larger leaves

AtGRF2 7-2 ... overexpressors ... developed larger ... cotyledons

AtGRF1 4-7 ... overexpressors ... developed larger leaves

[Visit the PubMed Article](http://www.ncbi.nlm.nih.gov/pubmed/12974814)

**PubMed ID: 14623244**

Wang Y, Henriksson E, Söderman E, Henriksson KN, Sundberg E, Engström P - The Arabidopsis homeobox gene, ATHB16, regulates leaf development and the sensitivity to photoperiod in Arabidopsis

the leaves of 35S::antiATHB16 plants grown under LD were larger than wild-type leaves

[Visit the PubMed Article](http://www.ncbi.nlm.nih.gov/pubmed/14623244)

**PubMed ID: 15073153**

Ueda M, Matsui K, Ishiguro S, Sano R, Wada T, Paponov I, Palme K, Okada K - The HALTED ROOT gene encoding the 26S proteasome subunit RPT2a is essential for the maintenance of Arabidopsis meristems

In mature embryos of the mutant, both the size and cell arrangement of the SAM were normal, and cell layers were clearly observed (Fig. 2E,F; black lines). However, the SAM was enlarged and the cell layers were disrupted in 8-day-old mutant seedlings, possibly owing to their irregular cell division planes (Fig. 2H, arrowheads). These results indicate that hlr mutants form a SAM of normal structure during embryogenesis, but fail to maintain its cellular organization after germination

[Visit the PubMed Article](http://www.ncbi.nlm.nih.gov/pubmed/15073153)

**PubMed ID: 15086805**

Asano T, Yoshioka Y, Kurei S, Sakamoto W, Machida Y; Sodmergen - A mutation of the CRUMPLED LEAF gene that encodes a protein localized in the outer envelope membrane of plastids affects the pattern of cell division, cell differentiation, and plastid division in Arabidopsis

crl mutant ... chloroplasts ... were extremely enlarged compared to those of the wild type

[Visit the PubMed Article](http://www.ncbi.nlm.nih.gov/pubmed/15086805)

**PubMed ID: 15319482**

Nam KH, Li J - The Arabidopsis transthyretin-like protein is a potential substrate of BRASSINOSTEROID-INSENSITIVE 1

the two ttl mutants were bigger than the wild-type plants of the same developmental age with larger rosette leaves

[Visit the PubMed Article](http://www.ncbi.nlm.nih.gov/pubmed/15319482)

**PubMed ID: 15937226**

Clay NK, Nelson T - The recessive epigenetic swellmap mutation affects the expression of two step II splicing factors required for the transcription of the cell proliferation gene STRUWWELPETER and for the timing of cell cycle arrest in the Arabidopsis leaf

in ANT overexpression plants; instead, final organ size was increased

smp ... leaf primordia ... sections revealed large intercellular spaces developing within the organ

[Visit the PubMed Article](http://www.ncbi.nlm.nih.gov/pubmed/15937226)

**PubMed ID: 15960614**

Okushima Y, Mitina I, Quach HL, Theologis A - AUXIN RESPONSE FACTOR 2 (ARF2): a pleiotropic developmental regulator

Homozygous plants of the ... arf2-8 have ... large ... rosette leaves

Homozygous plants of the ... arf2-7 ... have ... large ... rosette leaves

ARF2-RNAi lines ... have ... large ... rosette leaves

arf2-6 mutant seedlings have significantly enlarged cotyledons under ... Rc ... conditions despite more elongated hypocotyls

Homozygous plants of the arf2-6 ... have ... large ... rosette leaves

arf2-6 plants have slightly enlarged cotyledons

arf2-6 mutant seedlings have significantly enlarged cotyledons under ... FRc conditions despite more elongated hypocotyls

[Visit the PubMed Article](http://www.ncbi.nlm.nih.gov/pubmed/15960614)

**PubMed ID: 15960617**

Horiguchi G, Kim GT, Tsukaya H - The transcription factor AtGRF5 and the transcription coactivator AN3 regulate cell proliferation in leaf primordia of Arabidopsis thaliana

AN3 ... overexpressers ... developed leaves that were 20–30% larger than those of the wild type

AtGRF5 overexpressers ... developed leaves that were 20–30% larger than those of the wild type

[Visit the PubMed Article](http://www.ncbi.nlm.nih.gov/pubmed/15960617)

**PubMed ID: 16115070**

Delessert C, Kazan K, Wilson IW, Van Der Straeten D, Manners J, Dennis ES, Dolferus R - The transcription factor ATAF2 represses the expression of pathogenesis-related genes in Arabidopsis

In plants transformed with the 35S:ATAF2 construct, we observed ... an increased leaf size

[Visit the PubMed Article](http://www.ncbi.nlm.nih.gov/pubmed/16115070)

**PubMed ID: 16155177**

Teper-Bamnolker P, Samach A - The flowering integrator FT regulates SEPALLATA3 and FRUITFULL accumulation in Arabidopsis leaves

Pro35S:FT/TFT plants grown at 12°C had much larger leaves with no sign of curling

[Visit the PubMed Article](http://www.ncbi.nlm.nih.gov/pubmed/16155177)

**PubMed ID: 16222298**

Grigg SP, Canales C, Hay A, Tsiantis M - SERRATE coordinates shoot meristem function and leaf axial patterning in Arabidopsis

se-2 and se-3 plants show enlarged embryonic SAMs

se-3 mutants develop a substantially hypertrophic vegetative SAM

[Visit the PubMed Article](http://www.ncbi.nlm.nih.gov/pubmed/16222298)

**PubMed ID: 16284709**

Horiguchi G, Ferjani A, Fujikura U, Tsukaya H - Coordination of cell proliferation and cell expansion in the control of leaf size in Arabidopsis thaliana

transgenic plants overexpressing transcription fac- tors such as AINTEGUMENTA (ANT) ... develop larger leaves than the wild type

transgenic plants overexpressing transcription fac- tors such as ... ANGUSTIFOLIA3/GRF-INTERACTING FACTOR1 [AN3/GIF1]), develop larger leaves than the wild type

known large- leaf mutants ... sgs3

known large- leaf mutants ... arf2

transgenic plants overexpressing transcription fac- tors such as ... GROWTH REGULATING FACTOR5 (AtGRF5) ... develop larger leaves than the wild type

known large- leaf mutants ... ron2

large leaves ... grandifolia1-1D (gra1-1D)

develop larger leaves than the wild type ... over- expression of ... AtGRF2

known large- leaf mutants ... ein3

known large- leaf mutants ... sgs2

develop larger leaves than the wild type ... over- expression of AtGRF1

known large- leaf mutants ... ttl

known large- leaf mutants ... ein2

known large- leaf mutants ... cpl1

known large- leaf mutants ... rev

[Visit the PubMed Article](http://www.ncbi.nlm.nih.gov/pubmed/16284709)

**PubMed ID: 16292465**

Beemster GT, Vercruysse S, De Veylder L, Kuiper M, Inzé D - The Arabidopsis leaf as a model system for investigating the role of cell cycle regulation in organ growth

Arath;KRP2 overexpressing ... leaf ... cells became much larger in the transgenic plants than in the wild type. It is noteworthy that this compensation had already occurred at the earliest sampling time when cells were still actively dividing, and that these early differences in relative size were maintained throughout the further development of these leaves

[Visit the PubMed Article](http://www.ncbi.nlm.nih.gov/pubmed/16292465)

**PubMed ID: 16339187**

Schruff MC, Spielman M, Tiwari S, Adams S, Fenby N, Scott RJ - The AUXIN RESPONSE FACTOR 2 gene of Arabidopsis links auxin signalling, cell division, and the size of seeds and other organs

mnt ... leaves are larger

[Visit the PubMed Article](http://www.ncbi.nlm.nih.gov/pubmed/16339187)

**PubMed ID: 16531491**

Cnops G, Neyt P, Raes J, Petrarulo M, Nelissen H, Malenica N, Luschnig C, Tietz O, Ditengou F, Palme K, Azmi A, Prinsen E, Van Lijsebettens M - The TORNADO1 and TORNADO2 genes function in several patterning processes during early leaf development in Arabidopsis thaliana

lateral meristems of the leaf primordia ... in trn2-1 ... contained vacuolated cells

[Visit the PubMed Article](http://www.ncbi.nlm.nih.gov/pubmed/16531491)

**PubMed ID: 16600425**

Duguay J, Jamal S, Liu Z, Wang TW, Thompson JE - Leaf-specific suppression of deoxyhypusine synthase in Arabidopsis thaliana enhances growth without negative pleiotropic effects

phenotype of the leaf-specific, DHS-suppressed transgenic plants ... was an enhancement in rosette leaf size. At 4 weeks of age just before the plants had begun to bolt, the rosette leaves for transgenic lines 101B, 104A and 116B were visibly larger than those of corresponding WT and empty-binary vector plants, and this size difference was maintained through weeks 5 and 6 by which time the plants had flowered and produced siliques (Fig. 7)

[Visit the PubMed Article](http://www.ncbi.nlm.nih.gov/pubmed/16600425)

**PubMed ID: 16698900**

Hricová A, Quesada V, Micol JL - The SCABRA3 nuclear gene encodes the plastid RpoTp RNA polymerase, which is required for chloroplast biogenesis and mesophyll cell proliferation in Arabidopsis

Some chloroplasts of the sca3-2 mutant displayed enlarged thylakoid lamellas

[Visit the PubMed Article](http://www.ncbi.nlm.nih.gov/pubmed/16698900)

**PubMed ID: 16740150**

Skirycz A, Reichelt M, Burow M, Birkemeyer C, Rolcik J, Kopka J, Zanor MI, Gershenzon J, Strnad M, Szopa J, Mueller-Roeber B, Witt I - DOF transcription factor AtDof1

we found opposite effects in mature RNAi–OBP2 plants, which were slightly larger due to increased leaf size

[Visit the PubMed Article](http://www.ncbi.nlm.nih.gov/pubmed/16740150)

**PubMed ID: 16824178**

Hu Y, Poh HM, Chua NH - The Arabidopsis ARGOS-LIKE gene regulates cell expansion during organ growth

at least ... nine ARL-OE lines displayed ... larger ... leaves

we generated transgenic plants ... overexpressing ARL cDNA (ARL-OE) ... at least ... nine ARL-OE lines displayed ... larger cotyledons

[Visit the PubMed Article](http://www.ncbi.nlm.nih.gov/pubmed/16824178)

**PubMed ID: 16916932**

White DW - PEAPOD regulates lamina size and curvature in Arabidopsis

In Δppd plants ... cotyledon laminae were larger

In Δppd plants, leaf ... laminae ... were larger

[Visit the PubMed Article](http://www.ncbi.nlm.nih.gov/pubmed/16916932)

**PubMed ID: 17041028**

Galichet A, Gruissem W - Developmentally controlled farnesylation modulates AtNAP1;1 function in cell proliferation and cell expansion during Arabidopsis leaf development

9-d-old Atnap1;1-1 and Atnap1;1-2 cotyledons were enlarged

leaves ... were enlarged in plants expressing AtNAP1;1C369S

cotyledons ... were enlarged in plants expressing AtNAP1;1C369S

[Visit the PubMed Article](http://www.ncbi.nlm.nih.gov/pubmed/17041028)

**PubMed ID: 17351828**

Chiu WH, Chandler J, Cnops G, Van Lijsebettens M, Werr W - Mutations in the TORNADO2 gene affect cellular decisions in the peripheral zone of the shoot apical meristem of Arabidopsis thaliana

A typical feature of line 3010 mutant seedlings was an enlarged apical dome

[Visit the PubMed Article](http://www.ncbi.nlm.nih.gov/pubmed/17351828)

**PubMed ID: 17397538**

Eyüboglu B, Pfister K, Haberer G, Chevalier D, Fuchs A, Mayer KF, Schneitz K - Molecular characterisation of the STRUBBELIG-RECEPTOR FAMILY of genes encoding putative leucine-rich repeat receptor-like kinases in Arabidopsis thaliana

transgenic wild-type Col plants ectopically expressing SRF4 using the 35S promoter of cauliflower mosaic virus [49] exhibited leaves of increased size

[Visit the PubMed Article](http://www.ncbi.nlm.nih.gov/pubmed/17397538)

**PubMed ID: 18061566**

Anastasiou E, Kenz S, Gerstung M, MacLean D, Timmer J, Fleck C, Lenhard M - Control of plant organ size by KLUH/CYP78A5-dependent intercellular signaling

moderately increasing KLU activity in its endogenous expression domain (Figures S2F and S2G) causes overgrowth of leaves

[Visit the PubMed Article](http://www.ncbi.nlm.nih.gov/pubmed/18061566)

**PubMed ID: 18317696**

Kwon YR, Lee HJ, Kim KH, Hong SW, Lee SJ, Lee H - Ectopic expression of Expansin3 or Expansinbeta1 causes enhanced hormone and salt stress sensitivity in Arabidopsis

The size of the AtEXP3-OX leaves exhibit approximately larger than those of the wild type seedlings by 1.5-fold

[Visit the PubMed Article](http://www.ncbi.nlm.nih.gov/pubmed/18317696)

**PubMed ID: 18483219**

Li Y, Zheng L, Corke F, Smith C, Bevan MW - Control of final seed and organ size by the DA1 gene family in Arabidopsis thaliana

eod1-1da1-1 double mutant exhibited substantially larger seed and organ size and longer life span than da1-1

da1-1 plants have ... large leaves

[Visit the PubMed Article](http://www.ncbi.nlm.nih.gov/pubmed/18483219)

**PubMed ID: 18485060**

Liu Y, Wang F, Zhang H, He H, Ma L, Deng XW - Functional characterization of the Arabidopsis ubiquitin-specific protease gene family reveals specific role and redundancy of individual members in development

UBP15 over-expression lines revealed larger overall stature of the plants as well as larger rosette leaves

UBP15 over-expression lines revealed larger overall stature of the plants

[Visit the PubMed Article](http://www.ncbi.nlm.nih.gov/pubmed/18485060)

**PubMed ID: 18492871**

Wang JW, Schwab R, Czech B, Mica E, Weigel D - Dual effects of miR156-targeted SPL genes and CYP78A5/KLUH on plastochron length and organ size in Arabidopsis thaliana

increased shoot apical meristem size in clv1 mutants

[Visit the PubMed Article](http://www.ncbi.nlm.nih.gov/pubmed/18492871)

**PubMed ID: 18816164**

Schommer C, Palatnik JF, Aggarwal P, Chételat A, Cubas P, Farmer EE, Nath U, Weigel D - Control of jasmonate biosynthesis and senescence by miR319 targets

tcp2 tcp4 double mutants showed a further increase in leaf size

among plants that overexpressed miR319a from a constitutive 35S promoter, weak lines had bigger, but not crinkly leaves, similar to the tcp single knockout plants

TCP2 ... Loss-of-function ... had ... slightly enlarged leaves

TCP10 ... Loss-of-function ... had ... slightly enlarged leaves

TCP4 ... Loss-of-function ... had ... slightly enlarged leaves

[Visit the PubMed Article](http://www.ncbi.nlm.nih.gov/pubmed/18816164)

**PubMed ID: 19074624**

Larson-Rabin Z, Li Z, Masson PH, Day CD - FZR2/CCS52A1 expression is a determinant of endoreduplication and cell expansion in Arabidopsis

Expression of the FZR2 cDNA with the constitutive cauliflower mosaic virus 35S promoter (denoted OE ... produced trichomes with ... enlarged nuclei

the larger OE trichomes possessed nuclei that seemed to have spread out even more (Fig. 4, D–G

Class II (OE II) plants were slightly larger

[Visit the PubMed Article](http://www.ncbi.nlm.nih.gov/pubmed/19074624)

**PubMed ID: 19143999**

Hong Y, Devaiah SP, Bahn SC, Thamasandra BN, Li M, Welti R, Wang X - Phospholipase D epsilon and phosphatidic acid enhance Arabidopsis nitrogen signaling and growth

Enhanced growth in PLDε-OE plants was also observed under less well-fertilized conditions

PLDε-OE ... increases in leaf size

The cell size of OE leaves was larger

[Visit the PubMed Article](http://www.ncbi.nlm.nih.gov/pubmed/19143999)

**PubMed ID: 19200151**

Guan H, Kang D, Fan M, Chen Z, Qu LJ - Overexpression of a new putative membrane protein gene AtMRB1 results in organ size enlargement in Arabidopsis

AtMRB1 overexpressor plants ... exhibited enlarged organ sizes

AtMRB1 overexpressor plants were larger

[Visit the PubMed Article](http://www.ncbi.nlm.nih.gov/pubmed/19200151)

**PubMed ID: 19216774**

Schröder F, Lisso J, Lange P, Müssig C - The extracellular EXO protein mediates cell expansion in Arabidopsis leaves

BR application resulted in larger cotyledons

[Visit the PubMed Article](http://www.ncbi.nlm.nih.gov/pubmed/19216774)

**PubMed ID: 19321709**

Kurepa J, Wang S, Li Y, Zaitlin D, Pierce AJ, Smalle JA - Loss of 26S proteasome function leads to increased cell size and decreased cell number in Arabidopsis shoot organs

palisade ... cells of ... rpn12a-1 were enlarged at all stages of cotyledon development

3-d-old light-grown rpt2a mutants had enlarged cotyledons

[Visit the PubMed Article](http://www.ncbi.nlm.nih.gov/pubmed/19321709)

**PubMed ID: 19500299**

Sonoda Y, Sako K, Maki Y, Yamazaki N, Yamamoto H, Ikeda A, Yamaguchi J - Regulation of leaf organ size by the Arabidopsis RPT2a 19S proteasome subunit

Trichome nuclear size was determined by measuring two-dimensional images of DAPI-stained trichomes, and revealed that the size of rpt2a mutant trichome nuclei are, on average, larger than those of the wild type

Trichomes of the rpt2a-2 mutant were larger than the wild type

To evaluate the correlation between the enlarged leaves and cell nuclei size, the nuclei of epidermal cells from the fifth rosette leaves were observed by DAPI staining. Consistent with the increase in endoreduplication, larger nuclei were observed in the larger cells of rpt2a-2 compared with the wild type

The rpt2a-2 mutant contained greatly expanded first and seventh rosette leaves

rpt2a-2 mutant ... displayed a phenotype of enlarged rosette leaves

the rpt2a-1 mutant displayed a phenotype of enlarged rosette leaves

[Visit the PubMed Article](http://www.ncbi.nlm.nih.gov/pubmed/19500299)

**PubMed ID: 19508432**

Horiguchi G, Gonzalez N, Beemster GT, Inzé D, Tsukaya H - Impact of segmental chromosomal duplications on leaf size in the grandifolia-D mutants of Arabidopsis thaliana

we selected ... semi-dominant ... gra3-D ... mutants had an enlarged leaf phenotype

Heterozygous ... gra2-D ... mutants ... had a leaf size intermediate between that of wild-type and homozygous mutants

Heterozygous ... gra3-D mutants ... had a leaf size intermediate between that of wild-type and homozygous mutants

gra1-D ... increase in leaf size was less evident in leaves formed at later developmental stages

gra2-D ... increase in leaf size was less evident in leaves formed at later developmental stages

we introduced a genomic fragment including the promoter region and the 3′ untranslated region of CYCD3;1 into the wild-type (designated CYCD3;1 ... resulting independent CYCD3;1+ lines had slightly enlarged leaves

gra3-D ... increase in leaf size was less evident in leaves formed at later developmental stages

Heterozygous gra1-D ... mutants ... had a leaf size intermediate between that of wild-type and homozygous mutants

we selected ... semi-dominant ... gra2-D ... mutants had an enlarged leaf phenotype

we selected ... semi-dominant ... gra1-D ... mutants had an enlarged leaf phenotype

[Visit the PubMed Article](http://www.ncbi.nlm.nih.gov/pubmed/19508432)

**PubMed ID: 20023165**

Rodriguez RE, Mecchia MA, Debernardi JM, Schommer C, Weigel D, Palatnik JF - Control of cell proliferation in Arabidopsis thaliana by microRNA miR396

Plants expressing rGRF2 in a wild-type background had larger leaves

[Visit the PubMed Article](http://www.ncbi.nlm.nih.gov/pubmed/20023165)

**PubMed ID: 20040585**

Ichihashi Y, Horiguchi G, Gleissberg S, Tsukaya H - The bHLH transcription factor SPATULA controls final leaf size in Arabidopsis thaliana

To determine how SPT limits leaf size, we compared the time course of leaf development between spt-11 and the wild type based on the DAS (Fig. 4). At 3 DAS, leaf primordia in spt-11 were similar to those of the wild type in terms of their shape (Fig. 4A, D) and size (leaf length, wild type, 81.3 ± 16.3 μm; spt-11, 91.4 ± 7.3 μm; no significant difference by Student’s t-test, P < 0.05). However, the spt-11 leaf primordia were about 1.5-fold larger than wild-type leaf primordia at 5 DAS (Fig. 4B, E, G) and such a difference was maintained throughout subsequent development (Fig. 4C, F). Given that the detectable cause of the large leaf phenotype in spt-11 can be traced back to 5 DAS but not to 3 DAS, a critical event influencing the final leaf size must take place during this developmental window

[Visit the PubMed Article](http://www.ncbi.nlm.nih.gov/pubmed/20040585)

**PubMed ID: 20460583**

Gonzalez N, De Bodt S, Sulpice R, Jikumaru Y, Chae E, Dhondt S, Van Daele T, De Milde L, Weigel D, Kamiya Y, Stitt M, Beemster GT, Inzé D - Increased leaf size: different means to an end

transgenic lines overexpressing ... GRF5 ... produced significantly enlarged leaves ... GRF5 produced enlarged older leaves (leaves ... 1–4

transgenic lines overexpressing ... AVP1 ... produced significantly enlarged leaves ... In the AVP1 line, most leaves were larger than those of the wild-type plants, so that the whole rosette area had significantly increased

transgenic lines overexpressing ... JAW ... produced significantly enlarged leaves ... for JAW, we only observed a slight increase of the size of the first leaves produced, while the later leaves and the rosette as a whole were much reduced. The large leaf size reported previously (Palatnik et al., 2003) only becomes apparent when plants are grown for a prolonged period, well after the time that the Columbia-0 (Col-0) wild type has started flowering

transgenic lines overexpressing ... BRI1 ... produced significantly enlarged leaves ... BRI1 ... produced enlarged older leaves (leaves 1–5

transgenic lines overexpressing ... GA20OX1 ... produced significantly enlarged leaves ... overexpression of GA20OX1 increased the size of the younger leaves (leaves 4–10) but not the older ones

[Visit the PubMed Article](http://www.ncbi.nlm.nih.gov/pubmed/20460583)

**PubMed ID: 20628155**

Sarojam R, Sappl PG, Goldshmidt A, Efroni I, Floyd SK, Eshed Y, Bowman JL - Differentiating Arabidopsis shoots from leaves by combined YABBY activities

Epidermal cells of YABBY quadruple mutant leaves are uniformly large compared with the wild type

[Visit the PubMed Article](http://www.ncbi.nlm.nih.gov/pubmed/20628155)

**PubMed ID: 21143677**

Abbasi N, Kim HB, Park NI, Kim HS, Kim YK, Park YI, Choi SB - APUM23, a nucleolar Puf domain protein, is involved in pre-ribosomal RNA processing and normal growth patterning in Arabidopsis

the size of the nucleolus in the apum23-1 mutant was bigger than that in the Col-3 wild-type background ... We crossed the apum23-1 mutant with a 35S:AtFib2-GFP transgenic plant and obtained homozygous lines for the apum23-1 mutant that expressed AtFib2–GFP protein. As a control, we used the same parental line (35S:AtFib2-GFP) that was used for crossing with apum23-1, to minimize differences in expression of the fusion protein. Confocal images of 3-week-old leaves were obtained for wild-type plants (Figure 4l,m) and apum23-1 plants expressing AtFib2–GFP (Figure 4n,o). Because the nucleolus is a highly dynamic structure, we measured the area of the nucleoli in which AtFib2–GFP was found to localize. Consistent with the data obtained from electron microscopy images, apum23-1 plants had a greater percentage of larger nucleoli, while wild-type plants showed a higher frequency of smaller nucleoli

[Visit the PubMed Article](http://www.ncbi.nlm.nih.gov/pubmed/21143677)

**PubMed ID: 21214652**

Yu X, Li L, Zola J, Aluru M, Ye H, Foudree A, Guo H, Anderson S, Aluru S, Liu P, Rodermel S, Yin Y - A brassinosteroid transcriptional network revealed by genome-wide identification of BESI target genes in Arabidopsis thaliana

bes1-D chloroplasts have dramatically enlarged plastoglobules (PG) (Figure 6b,c). Plastoglobules are lipoprotein bodies that participate in a variety of metabolic pathways, primarily involving lipids, carotenoids and tocopherols (vitamin E)

[Visit the PubMed Article](http://www.ncbi.nlm.nih.gov/pubmed/21214652)

**PubMed ID: 21241390**

Raschke M, Boycheva S, Crèvecoeur M, Nunes-Nesi A, Witt S, Fernie AR, Amrhein N, Fitzpatrick TB - Enhanced levels of vitamin B(6) increase aerial organ size and positively affect stress tolerance in Arabidopsis

AtPDX1.1-2 OE lines ... the leaves of the mature rosette of the overexpressor lines were considerably larger than those of wild-type

AtPDX1.1-2 OE ... noted that all aerial organs were enlarged in the lines with more vitamin B6, i.e. stems, cauline leaves, flowers, siliques

AtPDX1.1 OE ... increase in the size of the cotyledons of the corresponding seedlings

AtPDX1.1 OE ... we noted that all aerial organs were enlarged in the lines with more vitamin B6, i.e. stems, cauline leaves, flowers, siliques

AtPDX1.1 OE ... the leaves of the mature rosette of the overexpressor lines were considerably larger than those of wild-type

AtPDX1.1-2 OE ... increase in the size of the cotyledons of the corresponding seedlings

[Visit the PubMed Article](http://www.ncbi.nlm.nih.gov/pubmed/21241390)

**PubMed ID: 21401745**

Ohbayashi I, Konishi M, Ebine K, Sugiyama M - Genetic identification of Arabidopsis RID2 as an essential factor involved in pre-rRNA processing

Hypocotyl explants of the rid2-1 mutant were inspected under a microscope, and the stele cells were found to be abnormally swollen during culture on CIM at 28°C (Figure 4a). In these cells, the nucleoli were extraordinarily large, and each nucleolus contained a prominent and very large nucleolar cavity (Figure 4a). This unusual structure of the nucleolus of rid2-1 was confirmed by using green fluorescent protein (GFP)-tagged NHP2 (NHP2:GFP) as a nucleolar protein marker (Figure 4b), and by double-staining with 2-{4-[amino(imino)methyl]phenyl}-1H-indole-6-carboximidamide (DAPI) and SYTO RNASelect (Molecular Probes, Invitrogen, http://www.invitrogen.com) (Figure 4c). Nucleolar vacuolation was also observed in various tissues of the rid2-1 seedlings that had been exposed to a temperature of 28°C (Figure 4d; Table S1). At 22°C, the effect of the rid2-1 mutation on the nucleolar structure was much weaker in all tissues, but was still detectable in comparison with the wild-type cells (Figure 4a,b,d; Table S1

[Visit the PubMed Article](http://www.ncbi.nlm.nih.gov/pubmed/21401745)

**PubMed ID: 21453984**

Köllmer I, Werner T, Schmülling T - Ectopic expression of different cytokinin-regulated transcription factor genes of Arabidopsis thaliana alters plant growth and development

Only strong expressing 35S:bHLH64 transgenic lines (35S:bHLH64-88, 35S:bHLH64-89) showed strong morphological differences compared to the wild type and were analyzed. These plants formed as heterozygotes a larger rosette with lanceolate leaves displaying serrated margins (not shown) and a gene dose-dependent reduced shoot height

[Visit the PubMed Article](http://www.ncbi.nlm.nih.gov/pubmed/21453984)

**PubMed ID: 21457262**

Feng G, Qin Z, Yan J, Zhang X, Hu Y - Arabidopsis ORGAN SIZE RELATED1 regulates organ growth and final organ size in orchestration with ARGOS and ARL

Compared with control lines, which contained an empty vector, all of 20 independently transgenic lines overexpressing OSR1 displayed visibly enlarged aerial organs, including leaves

Compared with control lines, which contained an empty vector, all of 20 independently transgenic lines overexpressing OSR1 displayed visibly enlarged aerial organs, including ... cotyledons

[Visit the PubMed Article](http://www.ncbi.nlm.nih.gov/pubmed/21457262)

**PubMed ID: 21457373**

Zhang Y, Zhang B, Yan D, Dong W, Yang W, Li Q, Zeng L, Wang J, Wang L, Hicks LM, He Z - Two Arabidopsis cytochrome P450 monooxygenases, CYP714A1 and CYP714A2, function redundantly in plant development through gibberellin deactivation

Many of the ELA1-RNAi/ela2 transgenic plants had ... enlarged rosettes ... 50% increase was observed for the sizes of ... rosette leaves

we generated an RNA interference (RNAi) transgene for ELA1, transformed the resulting transgene into the ela2 mutant, and generated many independent transgenic lines. Many of the ELA1-RNAi/ela2 transgenic plants had bigger cotyledons ... 30 ... % ... increase was observed for the sizes of cotyledons

[Visit the PubMed Article](http://www.ncbi.nlm.nih.gov/pubmed/21457373)

**PubMed ID: 21689171**

Chen MK, Hsu WH, Lee PF, Thiruvengadam M, Chen HI, Yang CH. - The MADS box gene, FOREVER YOUNG FLOWER, acts as a repressor controlling floral organ senescence and abscission in Arabidopsis

In the ethylene-insensitive response mutants, etr1-1 ... the plant ... size were larger than in the wild-type plants

In the ethylene-insensitive response mutants ... ein2-1 ... leaf size were larger than in the wild-type plants

In the ethylene-insensitive response mutants ... ein2-1 ... the plant ... size were larger than in the wild-type plants

In the ethylene-insensitive response mutants, etr1-1 ... leaf size were larger than in the wild-type plants

[Visit the PubMed Article](http://www.ncbi.nlm.nih.gov/pubmed/21689171)

**PubMed ID: 21711400**

Eloy NB, de Freitas Lima M, Van Damme D, Vanhaeren H, Gonzalez N, De Milde L, Hemerly AS, Beemster GT, Inzé D, Ferreira PC. - The APC/C subunit 10 plays an essential role in cell proliferation during leaf development

During the initial stages of leaf development, the leaf size between APC10OE and wild-type lines did not differ, but from day 10 onward, the difference became significant and, ultimately, the mature leaves of APC10OE were approximately 35% larger than those of the control, the difference first becoming visible at 11 DAS

To investigate the cellular basis of the observed phenotype, leaf development of APC10OE and wild-type plants grown in vitro was analyzed at the cellular level and as a function of time (De Veylder et al., 2001). In contrast to plants grown in soil, increased leaf size could already be observed in the first leaf pair of APC10OE plants

[Visit the PubMed Article](http://www.ncbi.nlm.nih.gov/pubmed/21711400)

**PubMed ID: 21781195**

Hashimura Y, Ueguchi C - The Arabidopsis MERISTEM DISORGANIZATION 1 gene is required for the maintenance of stem cells through the reduction of DNA damage

mdo1-1 shoot apex was ... enlarged laterally

[Visit the PubMed Article](http://www.ncbi.nlm.nih.gov/pubmed/21781195)

**PubMed ID: 21801253**

Zhang B, Chen HW, Mu RL, Zhang WK, Zhao MY, Wei W, Wang F, Yu H, Lei G, Zou HF, Ma B, Chen SY, Zhang JS - NIMA-related kinase NEK6 affects plant growth and stress response in Arabidopsis

At the flowering stage, the two NEK6-overexpressing lines exhibited ... larger rosette than Col

At the flowering stage, the two NEK6-overexpressing lines exhibited larger leaf ... than Col

[Visit the PubMed Article](http://www.ncbi.nlm.nih.gov/pubmed/21801253)

**PubMed ID: 21960139**

Irigoyen S, Karlsson PM, Kuruvilla J, Spetea C, Versaw WK - The sink-specific plastidic phosphate transporter PHT4;2 influences starch accumulation and leaf size in Arabidopsis

The large-leaf phenotype was also observed when plants were grown hydroponically

when plants were grown with a shorter photoperiod (8 h of light), fully expanded pht4;2 rosettes were notably larger than those of the wild type

[Visit the PubMed Article](http://www.ncbi.nlm.nih.gov/pubmed/21960139)

**PubMed ID: 22740614**

Goh HH, Sloan J, Dorca-Fornell C, Fleming A - Inducible repression of multiple expansin genes leads to growth suppression during leaf development

To investigate the cellular basis of these growth responses, a histological analysis was performed on induced and non-induced leaves (Table 1, Supp Fig 5). Contrary to the expected decrease in cell size suggested by the observed decrease in leaf lamina area (Fig. 4A, F), adaxial epidermal cells were actually significantly larger in the induced pOpON::amiREXP leaves than the controls and there was also a tendency for an increase ... in mesophyll cell size after repression of expansin gene expression

[Visit the PubMed Article](http://www.ncbi.nlm.nih.gov/pubmed/22740614)

**PubMed ID: 22864450**

Song JB, Huang SQ, Dalmay T, Yang ZM - Regulation of Leaf Morphology by MicroRNA394 and Its Target LEAF CURLING RESPONSIVENESS

The lcr plants display ... enlarged leaf blade

[Visit the PubMed Article](http://www.ncbi.nlm.nih.gov/pubmed/22864450)

**PubMed ID: 22869741**

Eloy NB, Gonzalez N, Van Leene J, Maleux K, Vanhaeren H, De Milde L, Dhondt S, Vercruysse L, Witters E, Mercier R, Cromer L, Beemster GT, Remaut H, Van Montagu MC, De Jaeger G, Ferreira PC, Inzé D - SAMBA, a plant-specific anaphase-promoting complex/cyclosome regulator is involved in early development and A-type cyclin stabilization

To investigate the effect of SAMBA inactivation at later stages of vegetative development, we analyzed leaf growth kinematically ... the leaf size of the first leaf pair of samba plants was already larger during the initial stages of development (Fig. 3A, Inset). At 4 DAS, the size of the samba leaf initials was on average 94% larger than that of the WT (Table S2), but, during further development, it became relatively less pronounced and at maturity (24 DAS) had increased ∼36% (Fig. 3A

Interestingly, the analysis of samba showed an increased seed size [up to 143% of the wild type (WT)] (Fig. 2A and Fig. S2A) and embryo size

To investigate the effect of SAMBA inactivation at later stages of vegetative development, we analyzed leaf growth kinematically ... the leaf size of the first leaf pair of samba plants was already larger during the initial stages of development ... Fig. 3A, Inset). At 4 DAS, the size of the samba leaf initials was on average 94% larger than that of the WT (Table S2

Additionally, samba rosettes were visibly larger

[Visit the PubMed Article](http://www.ncbi.nlm.nih.gov/pubmed/22869741)

**PubMed ID: 23289771**

Zhang H, Li L - SQUAMOSA promoter binding protein-like7 regulated microRNA408 is required for vegetative development in Arabidopsis

All leaves ... of the miR408-OX plants, are morphologically larger than those of wild-type plants

cotyledon of the miR408-OX plants, are morphologically larger than those of wild-type plants

[Visit the PubMed Article](http://www.ncbi.nlm.nih.gov/pubmed/23289771)

**PubMed ID: 23373882**

Tanaka Y, Nose T, Jikumaru Y, Kamiya Y - ABA inhibits entry into stomatal-lineage development in Arabidopsis leaves

the cyp707a1a3 mutant showed enlargement of cotyledon surface

enhanced expansion of GCs ... in abi1-1

enlargement of GCs ... in aba2-2 on day 10

increase in stomatal aperture size seen in aba2-2 on day 10

[Visit the PubMed Article](http://www.ncbi.nlm.nih.gov/pubmed/23373882)

**PubMed ID: 23573875**

Scofield S, Dewitte W, Nieuwland J, Murray JA - The Arabidopsis homeobox gene SHOOT MERISTEMLESS has cellular and meristem-organisational roles with differential requirements for cytokinin and CYCD3 activity

We compared the SAM of WT and STMoe seedlings, and found that the apical dome of the STMoe SAM is larger than WT but with no obvious changes in organisation

[Visit the PubMed Article](http://www.ncbi.nlm.nih.gov/pubmed/23573875)

**PubMed ID: 23581962**

Kim Y, Park S, Gilmour SJ, Thomashow MF - Roles of CAMTA transcription factors and salicylic acid in configuring the low-temperature transcriptome and freezing tolerance of Arabidopsis

sid2–1 plants were considerably larger than the WT plants after prolonged exposure to low temperature

[Visit the PubMed Article](http://www.ncbi.nlm.nih.gov/pubmed/23581962)

**PubMed ID: 23647236**

Cheng Y, Cao L, Wang S, Li Y, Shi X, Liu H, Li L, Zhang Z, Fowke LC, Wang H, Zhou Y - Downregulation of multiple CDK inhibitor ICK/KRP genes upregulates the E2F pathway and increases cell proliferation, and organ and seed sizes in Arabidopsis

ick1/2/6/7 ... The mean cotyledon sizes of 7-day-old quadruple ... mutants were significantly larger than those of single, double and triple mutants as well as the Wt

ick1/2/5/6/7 ... The mean cotyledon sizes of 7-day-old ... quintuple mutants were significantly larger than those of single, double and triple mutants as well as the Wt

[Visit the PubMed Article](http://www.ncbi.nlm.nih.gov/pubmed/23647236)

**PubMed ID: 23662592**

Gao Y, Liu H, An C, Shi Y, Liu X, Yuan W, Zhang B, Yang J, Yu C, Gao H - Arabidopsis FRS4/CPD25 and FHY3/CPD45 work cooperatively to promote the expression of the chloroplast division gene ARC5 and chloroplast division

cpd45 ... characterized by ... chloroplasts that are enlarged

cpd25 ... characterized by ... chloroplasts that are enlarged

[Visit the PubMed Article](http://www.ncbi.nlm.nih.gov/pubmed/23662592)

**PubMed ID: 24118612**

Li S, Zachgo S - TCP3 interacts with R2R3-MYB proteins, promotes flavonoid biosynthesis and negatively regulates the auxin response in Arabidopsis thaliana

developing bigger leaves ... p35S::TCP3SRDX-expressing plants

[Visit the PubMed Article](http://www.ncbi.nlm.nih.gov/pubmed/24118612)

**PubMed ID: 24151294**

Pineau B, Bourge M, Marion J, Mauve C, Gilard F, Maneta-Peyret L, Moreau P, Satiat-Jeunemaître B, Brown SC, De Paepe R, Danon A - The importance of cardiolipin synthase for mitochondrial ultrastructure, respiratory function, plant development, and stress responses in Arabidopsis

cls1 leaf cells ... giant mitochondria (roughly 28%) were observed, with a 2- to 6-µm-long axis

[Visit the PubMed Article](http://www.ncbi.nlm.nih.gov/pubmed/24151294)

**PubMed ID: 24399300**

Yi D, Alvim Kamei CL, Cools T, Vanderauwera S, Takahashi N, Okushima Y, Eekhout T, Yoshiyama KO, Larkin J, Van den Daele H, Conklin P, Britt A, Umeda M, De Veylder L - The Arabidopsis SIAMESE-RELATED cyclin-dependent kinase inhibitors SMR5 and SMR7 regulate the DNA damage checkpoint in response to reactive oxygen species

To address the role of the different SMR genes in DNA stress checkpoint regulation, the growth response to HU treatment of plants silenced for SMR5 ... was compared with that of control plants (Columbia-0 [Col-0]). No significant difference in leaf size was observed for plants grown under standard conditions. By contrast, when comparing plants grown for 3 weeks in the presence of HU, the leaves of the knockout plants SMR5KO ... were significantly larger than that of the control plants

in the presence of HU ... Within the SMR5KO SMR7KO double mutant, the reduction in leaf size and cell number was even less

[Visit the PubMed Article](http://www.ncbi.nlm.nih.gov/pubmed/24399300)

**PubMed ID: 24443518**

Vercruyssen L, Verkest A, Gonzalez N, Heyndrickx KS, Eeckhout D, Han SK, Jégu T, Archacki R, Van Leene J, Andriankaja M, De Bodt S, Abeel T, Coppens F, Dhondt S, De Milde L, Vermeersch M, Maleux K, Gevaert K, Jerzmanowski A, Benhamed M, Wagner D, Vandepoele K, De Jaeger G, Inzé D. - ANGUSTIFOLIA3 binds to SWI/SNF chromatin remodeling complexes to regulate transcription during Arabidopsis leaf development

Growth of AN3-GR plants on 25 µM DEX from germination onwards led to the development of larger ... leaves 1 and 2, compared with mock-treated transformants and DEX-treated control plants

Growth of AN3-GR plants on 25 µM DEX from germination onwards led to the development of larger cotyledons ... compared with mock-treated transformants and DEX-treated control plants

[Visit the PubMed Article](http://www.ncbi.nlm.nih.gov/pubmed/24443518)

**PubMed ID: 7555701**

Talbert PB, Adler HT, Parks DW, Comai L - The REVOLUTA gene is necessary for apical meristem development and for limiting cell divisions in the leaves and stems of Arabidopsis thaliana

leaves of rev-1 mutants grew abnormally large

The rosette leaves of rev-1 plants were not readily distinguishable from wild-type No-0 leaves prior to bolting. As bolting began, however, the youngest rosette leaves became abnormally large ... as they matured

[Visit the PubMed Article](http://www.ncbi.nlm.nih.gov/pubmed/7555701)

**PubMed ID: 9655815**

McConnell JR, Barton MK - Leaf polarity and meristem formation in Arabidopsis

the SAM of phb-1d/+ mutant embryos is enlarged relative to wild-type indicating that a stronger meristem promoting signal may be present in phb-1d mutant embryos (Fig. 8A,B

[Visit the PubMed Article](http://www.ncbi.nlm.nih.gov/pubmed/9655815)

**PubMed ID: 9694802**

Kim GT, Tsukaya H, Uchimiya H - The ROTUNDIFOLIA3 gene of Arabidopsis thaliana encodes a new member of the cytochrome P-450 family that is required for the regulated polar elongation of leaf cells

rot3-2 mutant had a clearly dis- tinct phenotype, having enlarged leaf blades

cotyledons of the rot3-2 mutant were slightly larger than those of the wild type

[Visit the PubMed Article](http://www.ncbi.nlm.nih.gov/pubmed/9694802)
