# Supplementary material for: LEAFDATA: a literature-curated database for Arabidopsis leaf development
Source: Plant Methods. 2016 Feb 15;12:15. doi: 10.1186/s13007-016-0115-9 (PMC4754890; doi:10.1186/s13007-016-0115-9)
Supplement: Supplementary file 2 — 10.1186/s13007-016-0115-9 Number of results retrieved by keywords search using different terms for large-leaf phenotype. [file 13007_2016_115_MOESM2_ESM.docx]

**Additional file 2 Table S1 Number of results retrieved by keywords search using different terms for large-leaf phenotype.**

| **Query terms** | **Number of results** |
| --- | --- |
| size_PATO:0000586 increased size_PATO:0000117 | 173 |
| large leaf | 253 |
| large leaves | 162 |
| large lea | 301 |
| big leaf | 18 |
| big leaves | 12 |
| big lea | 23 |
| increased leaf size | 373 |
| increased size leaves | 185 |
| increased size lea | 432 |
